# Optimize Design of Run-Flat Tires by Simulation and Experimental Research

**DOI:** 10.3390/ma14030474

**Published:** 2021-01-20

**Authors:** Huaqiao Liu, Yiren Pan, Huiguang Bian, Chuansheng Wang

**Affiliations:** College of Electromechanical Engineering, Qingdao University of Science and Technology, Qingdao 266061, China; CM0004@tta-solution.com (H.L.); Bianhuiguang@163.com (H.B.)

**Keywords:** insert rubber, simulation, heat accumulation, stress distribution, run-flat tire

## Abstract

In this study, the two key factors affecting the thermal performance of the insert rubber and stress distribution on the tire sidewall were analyzed extensively through various performance tests and simulations to promote the development of run-flat tires. Four compounds and two structures of insert rubber were designed to investigate the effects of heat accumulation and stress distribution on durability testing at zero pressure. It was concluded that the rigidity and tensile strength of the compound were negatively correlated with temperature. The deformation was a key factor that affects energy loss, which could not be judged solely by the loss factor. The stress distribution, however, should be considered in order to avoid early damage of the tire caused by stress concentration. On the whole, the careful balance of mechanical strength, energy loss, and structural rigidity was the key to the optimal development of run-flat tires. More importantly, the successful implementation of the simulations in the study provided important and useful guidance for run-flat tire development.

## 1. Introduction

Deflated tires on vehicles can potentially have serious safety implications to both the occupants in the vehicle as well to other road users. Statistically, approximately 70% of traffic accidents on highways were caused by deflated tires, and the mortality rate of riding on a flat tire was close to 100% at speeds of more than 160 km/h [1,2]. Tire blowout causes the instantaneous loss of tire pressure, leading to sidewall collapse and support to the tire. Subsequently, the center of gravity of the vehicle changes abruptly and the vehicle veers out of control. The designing of run-flat tires had undergone many different iterations of new designs since Chrysler and US Royal introduced a concept of run-flat tire in 1958. As early as 1976, several types of run-flat tires have been developed by tire makers in the world since its concept was introduced and the representative ones are the banded tire introduced [3]. Nowadays, the typical run-flat tires adopt a specially designed sidewall support structure, which was able to support the weight of the vehicle even in the event of lost pressure that the run-flat tire is able to run at zero internal pressure, fully loaded, for a maximum 80 km at 80 km/h. Contrary to the sidewall of conventional pneumatic tire, the insert rubbers of the sidewall reinforced run-flat tire makes the major driving performances such as the riding comfort and the rolling resistance worse than the conventional pneumatic tire. However, in consideration of safety, the advantages of running flat tire are gradually accepted by the market. In 2001, the 4th generation of the BMW 7 Series launched the market, and was the first model of mass-produced vehicles to be equipped with run-flat tires [4,5,6,7,8], which represents that the run-flat tire has officially entered the original market.

Compared to the traditional tire structure, the additional insert rubber and reinforcement of a run-flat tire inevitably changes the stress state of tire sidewalls during its running process. Many attempts have been made to investigate and optimize the properties of the tire self-supporting reinforced sidewall, based on the structure of the tire and insert rubber, including shape, thickness, compound, and flexion heat-generating properties of the insert rubber. Unlike traditional sidewall rubber, the properties of the insert rubber required not only higher rigidity for structural support, but also a higher degree of flexural resistance [9,10]. Nevertheless, these two properties were usually contradictory to each other and must be carefully balanced to achieve the optimum characteristics.

Generally, harder materials offer greater supporting capacity, but sacrifice flexural performance and were prone to higher levels of heat generation. However, these comparisons were usually based on the same types of deformation. If a softer rubber was to bear the same force, the result may be quite the opposite [11,12,13,14]. Therefore, the compound of the insert rubber must balance hardness and the flexibility in order to achieve optimum performance. Moreover, the sidewall of traditional tire was composed of several components, including beads, carcass piles, and sidewall chaffer. It had taken over a hundred years to develop a mature technical system.

Therefore, the rigidity of the insert rubber must match the rigidity of other structures of the tire sidewall to prevent the delamination of the internal components caused by driving with a deflated tire and early damage [15,16,17]. The development of a run-flat tire often requires significant amounts of resources to be invested for testing the tires in order to achieve the best performance. As such, this work employs mechanical analysis software to simulate and evaluate the stress state of the sidewall, in order to provide theoretical support for designing the structure and compound of the run-flat tire insert rubber. In addition, mechanical tests were carried out for two structures and four insert rubber compounds to further verify the correlation between the simulation results and tire performance.

## 2. Materials and Methods 

### 2.1. Preparation of the Tire and Simulation Model

**Figure 1:**
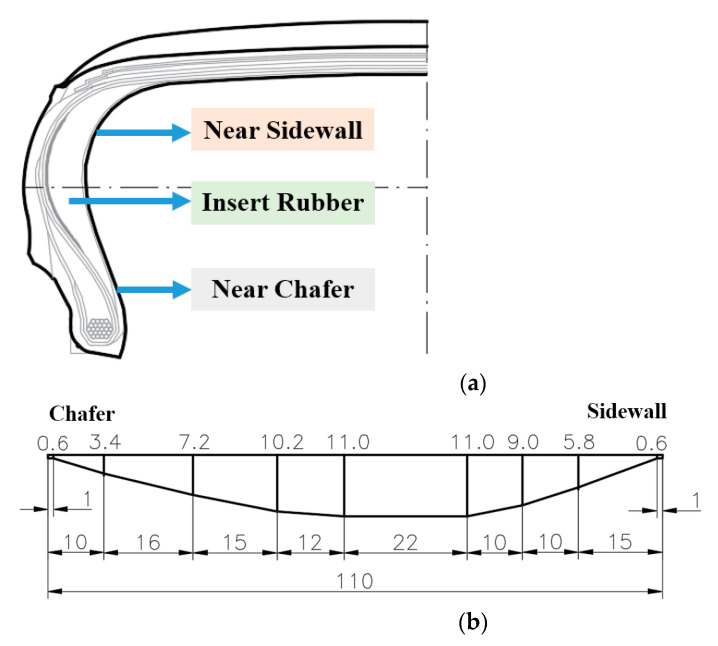

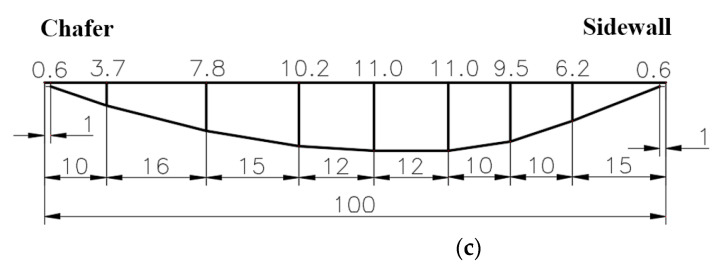
The structural designing of insert rubber (unit: mm). (**a**) Run-flat tire model; (**b**) B-1; (**c**) B-2. (unit: mm)

The two-dimensional (2D) model used in this simulation was the 245/45R18 96W model, and the structural designing of insert rubber was shown in Figure 1.

Figure 1 shows two structures of insert rubber which were designated as B-1 and B-2. B-1 ladder type had a large thickness transition range, while B-2 was a half-moon shape with a small thickness transition range. Compared with B-1, the total length of B-2 insert rubber was 10 mm smaller, the thickness of 11 mm at the middle points 5 and 6 was smaller, and the thickness at both ends was larger, which makes it more rigid and stress concentrated under flexural motion. In order to better research content of this paper, four groups of experimental formulae were designed with significant differences in heat generation and hardness which were used for simulation analysis and tire manufacturing with the combination of the two structures of insert rubber in Figure 1, and the simulation model scheme is shown in Table 1.

According to the Smithers FNF report of 225/45 R17 HP run-flat tire, the hardness of the support rubber of the run flat tire produced by the world-famous tire enterprises was generally between 71–78, and the rubber material in the formula was mainly cis-polybutadiene (BR) with natural rubber (NR), such as the support rubber of Bridgestone was NR/BR = 30/70, which was mainly considerate the high flexibility of BR. At the same time, other companies also selected natural rubber as the main raw material with cis-polybutadiene, such as Michelin insert rubber used NR/BR = 70/30 design, mainly considering the high physical and mechanical properties of nature rubber. Therefore, based on the two different rigid structures, we designed four groups of different hardness insert rubber formulations, the hardness gradients were 78/75/72/68, in which NR/BR = 70/30 was selected for A-2, and NR/BR = 30/70 was selected for the remaining three groups. Based on the previous development experience, too low hardness of insert rubber was not conducive to the tire’s durability under zero air pressure. Therefore, in this experiment, A-3 and A-4 were new combination designed and developed by us, which will be compared with the previous design. Under the structure of B-1, we only studied A-1 and A-2 system with higher hardness while the raw rubber design was just the opposite, so as to study the balance between raw rubber selection and hardness design.

Other elements of structural designing based on the tire simulation model were shown in Table 2.

#### Simulation Mathematical Models and Parameters

The description methods of mechanical properties of rubber materials can be roughly divided into two categories: One is the phenomenological description of rubber as a continuous medium, the other is the method based on thermodynamic statistics. At present, the polynomial models based on the theory of continuum mechanics are widely used in engineering such as Mooney- Rivlin model and Yeoh model. Because the stress-strain curve of rubber material had strong nonlinear characteristics, when the strain of rubber exceeds 20%, the material would show "hardening" characteristics, while the strain rate of insert rubber of run-flat tire was close to 25% at zero pressure. Therefore, in this case, the insert rubber appeared hardening. It was necessary to use the material constitutive model which can accurately described this characteristic to get the accurate simulation results. Mooney-Rivlin model could not reflect the "hardening" characteristics of rubber under large deformation, while the Yeoh model with a good fitting of rubber under large deformation could accurately reflect the state of the running tire at zero pressure.

The Yeoh model was used in ABAQUS simulation software to simulate run-flat tires. The Yeoh model was suitable for the large deformation behavior of filler-filled rubber. This model can predict the mechanical behavior of other deformation by fitting the parameters of deformation experimental data, and can reflect the s-shaped stress-strain curve under different deformation modes. The deformation range was also relatively wide, and the strain energy density function model was as follows:

(1) $$W=\overset{N}{\underset{i=1}{\sum }}C_{i0}(I_{1}-3{)}^{i}+\overset{N}{\underset{k=1}{\sum }}\frac{1}{d_{k}}(J-1{)}^{2k}$$

J was the volume ratio of after deformation and before deformation. When *N* = 3, the reduced polynomial is Yeoh model, which is a special form of reduced polynomial.

The typical binomial parameter form can be rewritten as:

(2) $$W=C_{10}\left(I_{1}-3\right)+C_{20}{\left(I_{1}-3\right)}^{2}$$

where *N*, $C_{ij}$, and $d_{k}$ were material constants, which were determined by material experiments, and the initial shear modulus was set as,

(3) $$\mu =2C_{10}$$

Rebar embedded unit was used to simulate the composite parts such as the crown layer, the band layer, and the body, and the rubber part was simulated by the incompressible C3D8H unit.

The Yeoh model construction equation in origin 8 was as follows:

(4) y=(((1+x)−(1+x)^(−2))x(2xc10+4xc20x((1+x)^2+2x(1+x)^(−1)−3)+6xc30x((1+x)^2       +2x(1+x)^(−1)−3)^2))+N

where *x* and *y* were the stress and strain values obtained based on the experimental data of rubber compound. The experimental data were imported into the data analysis software and fitted to obtain C10, C20, C30, and n. In the database of ABAQUS simulation analysis, the rubber material model was established by these four parameters, and finally used in the simulation analysis.

**Figure 2:**
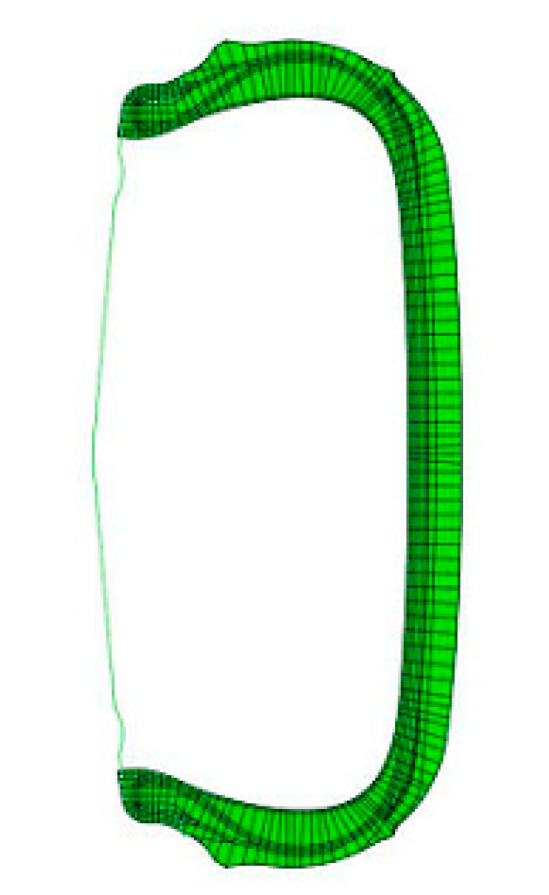
The mesh of tire model.

The mesh of tire model was shown in Figure 2.

In the process of tire and rim assembly, the global boundary condition X/Y axis was set immobility in the global coordinates, and only Z-axis motion was allowed to achieve contact between the bead and rim under inflation pressure. In the process of simulation. In the simulation process, the X-axis constraint was removed and the tire stress analysis under specific load and inflation pressure was started. Therefore, in order to simulate the stress of tire under zero pressure, the load is designed to be 80% of the tire load.

### 2.2. Materials

Natural rubber (NR): TSR20, Thailand 20# Standard Rubber; cis-polybutadiene (short for BR): BR9000, products of Beijing Yanshan Petrochemical Industry Co., Ltd.(Beijing, China); carbon black: N330.N550, products of Cabot, America (Cabot, Shanghai, China); zinc oxide (ZnO, white seal) were supplied by Hebei Hongtu Zinc Industry Co., Ltd.(Xingtai, China); stearic acid: Fengyi Oil Technology Co., Ltd. (Suzhou, China); N-(1,3-dimethylbutyl)-N0-phenyl-p-phenylenediamine (6PPD), 2,2,4-trimethyl-1,2-dihydroquinoline(TMQ), N-tert-butyl-2-benzothiazyl sulfenamide (TBBS), tetra(isobutyl) thioperoxydicarbamicacid (TIBTD), and insoluble sulfur (HSOT-20) were purchased from SAN’O Chemical Technology Co., Ltd. (Shanghai, China); paraffin wax was supplied by Shandong Yanggu Huatai Chemical Co., Ltd. (Liaocheng, China); treated distillate aromatic extract (TDAE) oil was obtained from H&R Group from Hamburg, Germany.

#### Preparation of the Insert Rubber Compounds

Table 3 was the compound compounds for experimental.

Four samples of insert rubber were prepared with the compounds shown in Table 3. Measured amounts of the components were mixed as a master batch in an industrial internal mixer (KOBELCO, BB430, Kobe, Japan). A three-stage mixing process for each compound was utilized to ensure the uniform dispersion of carbon black. At the first stage, the mixer was filled with rubber to a fill factor of 72%, during which the rubber was initially masticated for 15 s. Then, 75% of the carbon black was added into the mixture and mixed for 20 s or until the temperature of the master batch reached 115 °C. Next, oil was added (just for A-1) and mixed while the temperature of the master batch was below 145 °C. The dump temperature of the master batch compound was approximately 165 °C. In the second stage, the rest of the carbon black was added to the master batch 2 in the industrial internal mixer and mixed for 20 s. The master batch 2 compounds were discharged from the internal mixer at 155 °C. Finally, in stage three, the curatives TBBS, TIBTD, and sulfur, were mixed into the master batch 2 in an industrial internal mixer (Dalian Rubber & Plastics Machinery Co. Ltd., XM270, Dalian, China). The mixing was carried out at a rotor speed of 25 rpm and the mixer was filled to 67%. By controlling the time and temperature, the uniformity of rubber mixing can be ensured.

The detailed mixing procedures are specified in Table 4.

### 2.3. Characterization

The vulcanization of the rubber compounds was carried out in a hydraulic hot press at 150 °C based on the optimum cure time measured from the MDR (rotorless curemeter). The hardness was determined by using a Shore A durometer (Wallace H17A, UK) according to ISO 7619 Part 1. A universal testing machine (Instron 3366 series, Norfolk County, MA, USA) following (die type) was used to measure the tensile properties, while the dynamic properties were evaluated in tension mode using a dynamic mechanical thermal analyzer (DMTA: GABO Qualimeter Eplexor 25N, GABO, Germany). For the temperature sweep test, the test conditions were as follows: 7% static strain, 0.25% dynamic strain, frequency of 10 Hz, and 2 °C/min heating rate. The temperature was scanned from 40 °C to 160 °C.

## 3. Results and Discussion

### Vulcanization Properties

**Figure 3:**
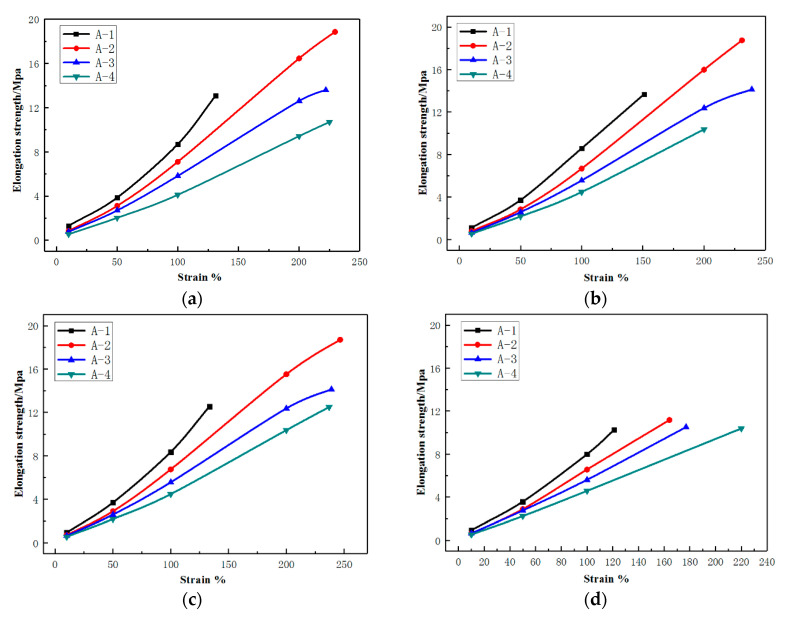

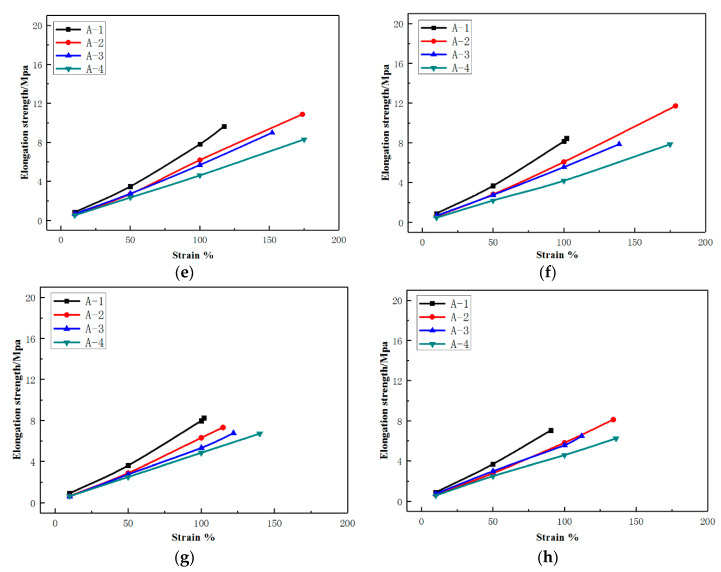
Temperature-dependent tensile properties of the vulcanizates. (**a**) tensile stress–strain plot at 23 °C; (**b**) tensile stress—strain plot at 30 °C; (**c**) tensile stress—strain plot at 40 °C; (**d**) tensile stress—strain plot at 60 °C; (**e**) tensile stress—strain plot at 80 °C; (**f**) tensile stress—strain plot at 100 °C; (**g**) tensile stress—strain plot at 120 °C; (**h**) tensile stress—strain plot at 140 °C. (Color figure can be viewed in the online issue).

The effects of temperature on the mechanical properties of the vulcanizates were illustrated in Figure 3, and the mechanical properties including tensile strength and elongation at break were summarized in Table 5. Generally, the higher the testing temperature, the lower the tensile strength and stress at certain elongation. This causes the damage resistance of the compound decrease with temperature increasing, revealing that both the thermal accumulation and the actual temperature directly affect the strength of the compound, and thus the tire performance. The results in Figure 3 also indicate that although the stress of formulation A-2 at a certain strain was lower than that of formulation A-1, its tensile strength and elongation at break were greater than those of A-1. Compared with A-3 and A-4, formulation A-2 had greater tensile strength and greater elongation at break. The reason for these differences may be the high content of NR in A-2, as the tensile crystallization property of NR made it have stronger mechanical strength than BR.

**Figure 4:**
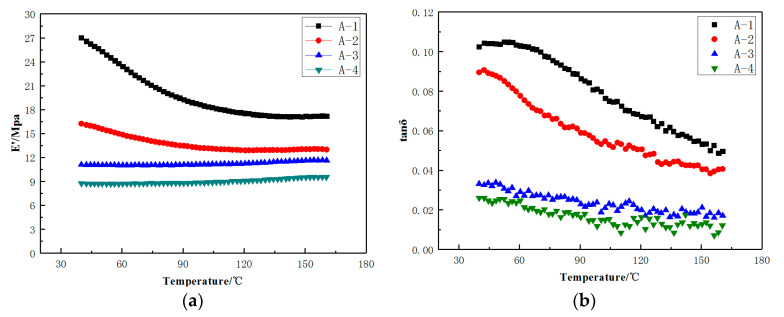
The dynamic mechanical characteristics of the vulcanizates. (**a**) Dynamic modulus (E’); (**b**) loss factor (tan δ).

The loss factor (tan δ) and the dynamic modulus (E’Mpa) of A-1 and A-4 in dynamic mechanical analysis temperature scanning were presented in Table 5. An analysis of the results indicates that the dynamic modulus tends to decrease with increasing test temperature. From Figure 4, the reduction in dynamic modulus can be seen to vary greatly at the two key temperature points of 100 °C and 160 °C, while only small variations were observed between those temperatures. In contrast, the loss factor was found to decrease with increasing temperature, and the rate of change was relatively consistent. As expected, the dynamic modulus and loss factor of formulation A-1, A-2, A-3, and A-4 decreased in turn, due to the inclusion N550 carbon black offering low heat generation and a lower filler content in formulation A-2, A-3, A-4.

Due to the varying loads across different tire applications, the performance of the four compounds cannot be accurately evaluated in practical conditions. The tests were performed based on ideal conditions of the same deformation and frequency. This was understandable as the tire was tested with the same loading. In other words, the tire compound was subjected to the same force.

To evaluate the performance of the compound under real-world conditions, the loss factor of the vulcanizates must take the actual deformation of the compound into consideration. Thus, energy dissipation △E was used to evaluate a strain period, and was expressed in Equation (5).

ΔE =πσ0 γ0sin δ ≈ πσ0γ0 tan δ(5)

where σ0 was the maximum amplitude of stress and γ0 was the maximum amplitude of strain.

Subsequently, four compounds of insert rubber with different modulus and heat-generation were designed to investigate the roles and impacts of deformation and loss factor of a tire which was shown in Figure 4.

Table 6 was the summarize of dynamic modulus (E’) and loss factor (tan δ) of the vulcanizates at various temperatures

Strictly speaking, the fatigue failure of rubber is a kind of mechanical process. The stress relaxation process produced in the material was often too late to complete in the deformation cycle under the repeated cyclic deformation of rubber. As a result, the internal generated stress could not be dispersed in time, and it might be concentrated in some defects (such as cracks, weak bonds, etc.), which caused fracture failure.

**Figure 5:**
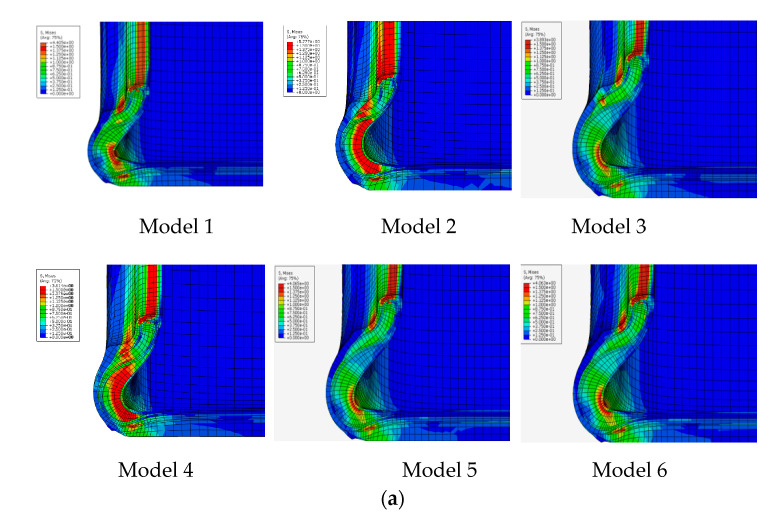

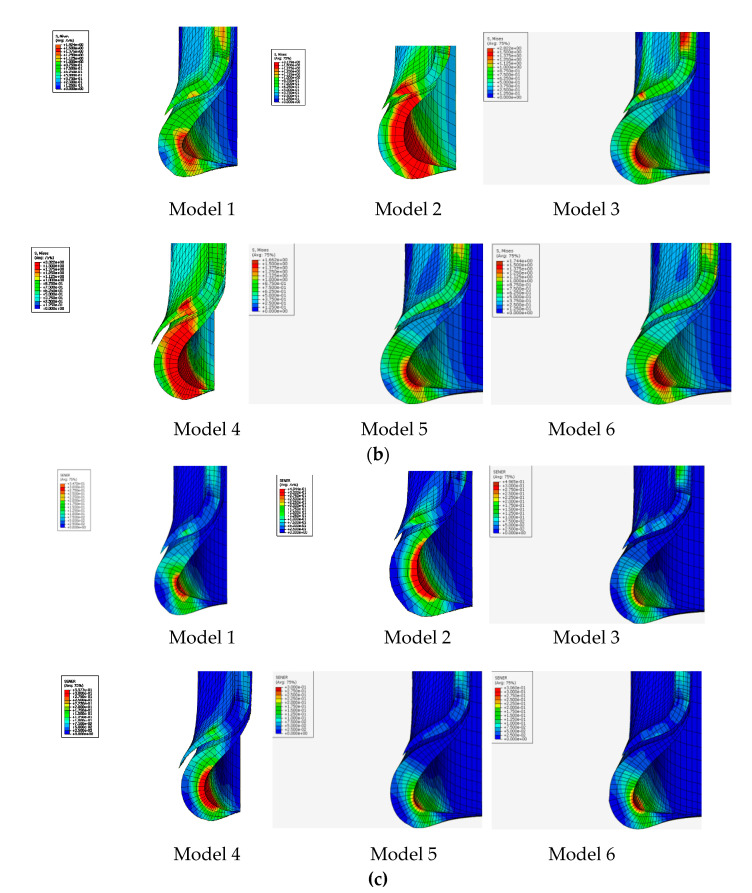
Simulation results of the models. (**a**) Stress distribution of tire section; (**b**) stress of bead apex and insert rubber of tire section; (**c**) strain energy density of bead apex and insert rubber of tire.

Figure 5 shows the simulation results of six tire models (size = 245/45/R18 96W) based on Table 1. The results of stress distribution of tire sidewall show that the stress was mainly concentrated in the insert compound in both the B-1 and B-2 structures which was shown in Figure 2. Surprisingly, using the A-1 compounds with the B-1 structure resulted in a more uniform stress distribution of the tire sidewall. This was because in the flexure test of the tire side adhesive, the stress was mainly concentrated on the insert support with high hardness, as shown in model 5. At the same time, compared with B-2, B-1 structure had a longer supporting compound, a larger amount of padding between the tread and the bead apex, and a better rigid transition with the tire sidewall, that was why the deformation of model 5 and model 6 on tire sidewall was greater than that of model 1, model 2, and model 3. Model 3 and model 4 used similar insert compound with soft hardness under the structure of B-2, the rigidity of the tire sidewall was weak, and the stress was concentrated on the weakest point of rigidity during flexion, which can be clearly seen from Figure 5a,b. The results of Table 6 also indicated that the actual stresses of the different compounds at the same part of the tire were different during the actual movement of the tire. This demonstrates why the loss factor cannot be directly used under the same deformation in the dynamic mechanical analysis test to evaluate the tire’s thermal performance.

Strain energy density of bead apex and insert rubber of tire were also shown in Figure 5c. Compared to model 5 and model 6, the strain energy density distribution of other models were more concentrated, suggesting a greater likelihood for the formation of a failure point.

Table 7 shows the summary of the analytical and quantitative results of the groups of analysis models. It is generally considered that the maximum stress and strain energy density at the bead apex rubber and support compound are the most important indexes related to the zero pressure durability of tire. The maximum stress and strain energy density of model 5 and model 6 under structure B-1 were smaller than those of other schemes, which indicates that the influence of structural rigidity is greater than that of the hardness of insert rubber. At the same time, under the same structure, the higher the hardness of the insert rubber, the smaller the stress and strain energy density, which can be found from the comparison of the previous four models.

**Figure 6:**
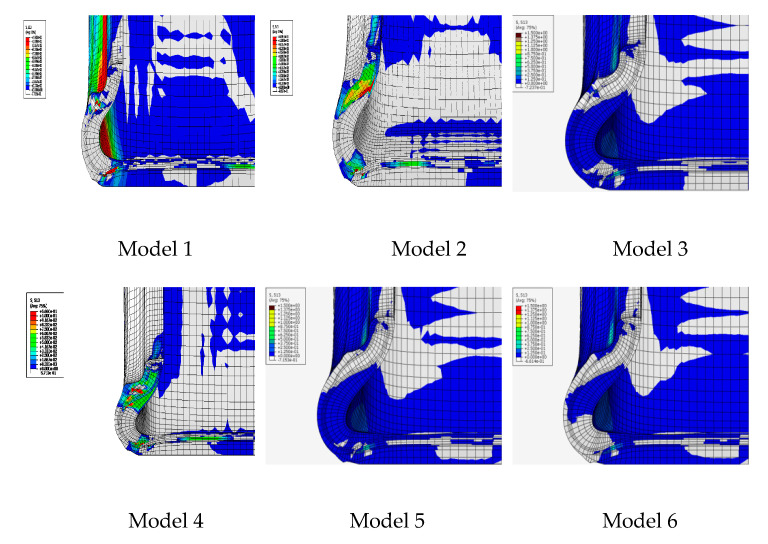
Shear stress distribution.

Figure 6 shows the shear stress distribution of the six models. There were no obvious benefits of reducing the length of the insert compound with respect to the direction of the shear force on each part of the tire sidewall, particularly between the insert rubber and bead apex. Delamination was easily induced by applying different directions of shear stress, causing damage points and premature damage. Compared with the first four models, model 5 and model 6 use B-1 structure, which had a longer contact surface with bead apex rubber, which effectively avoids the shear stress of relative slip between the two interfaces due to uneven stress. In model 3 and model 4, due to the use of insert rubber with lower hardness, the shear stress between the various parts of sidewall was small, mainly concentrated in the inner side of the tire at the center of sidewall. 

Based on the six simulation models, six actual tires under the structure of Table 2 were produced for testing, named as T-1, T-2, T-3, T-4, T-5, and T-6. Table 8 and Table 9 show the basic characteristics of the tires under a conventional static load and a zero-pressure static load. The static radius was most heavily influenced by the subsidence and subsidence rate under static load, and the structure of the insert rubber which provide structural rigidity to the tire. Further, it can be seen that under the same structure, the static load radius and sinkage of the tire were directly proportional to the rigidity of the insert rubber. Especially under the condition of zero pressure load, the stiffness of the insert rubber directly affects the static load radius and sinkage representing the tire deformation. Therefore, in comparison with T-5 and T-6, the greater deformation of T-1, T-2, T-3, and T-4 may lead to increases in heat generation.

**Figure 7:**
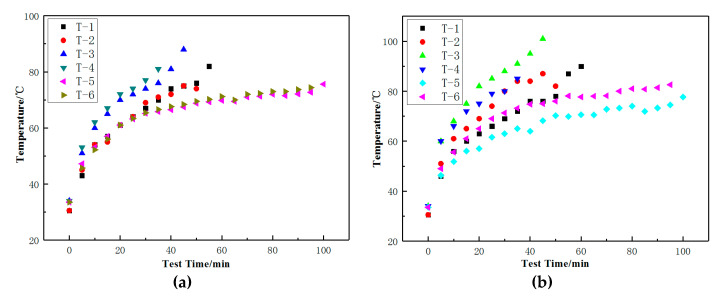
Temperature curve of tire testing. (**a**) Temperature curve of the tire sidewall; (**b**) temperature curve of tire bead.

The curves of temperature rise of the tire sidewall and bead during the zero-pressure durability test were shown in Figure 7. The sidewall and bead temperatures of T-1, T-2, T-3, and T-4 increase to a higher value over time, compared to T-5 and T-6. This was mainly due to the weaker structural rigidity of B-2 and the larger sinkage of the tire, in addition to higher energy loss according to the energy loss formula. Moreover, the results in Figure 7 reveal that although the loss factor of A-2 was lower than that of A-1, the difference in heat generation at the sidewalls of T-1 and T-2 was minimal, mainly due to the larger sinkage and the greater deformation of T-2, which led to higher energy loss. The comparison data of T-5 and T-6 with stronger tire structure can also prove this conclusion. The results further prove that the loss factor and deformation should be considered comprehensively in the energy loss of the rubber compound.

Table 10 summarizes the zero-pressure durability test results of the tires. It was evident that under the same rubber conditions, the results of the zero-pressure durability test were directly related to the rate of temperature rise of the tire sidewall. As the temperature increases, the resistance to damage of the compound decreases, which was clearly proven by the test results. At the same time, the stress distribution of tire sidewall was also found to directly affect results of the zero-pressure durability test. Although the difference of heat generated at the sidewall between T-1 and T-2 was minimal, an analysis of the strain energy density of bead apex and insert rubber of tire shown in Figure 5 suggested that the stress concentration in T-2 may lead to early tire failure. The test results of T-5 and T-6 can also prove this point. In addition, the zero pressure durability of T-5 and T-6 was better than that of T-1 and T-2 under the strong structural rigidity of B-1, which can also prove the effect of strain energy density. Compared with A-1 and A-2, the rigidity of A-3 and A-4 was lower. Although it can be seen from Table 6 that the loss factor of A-3 and A-4 was lower, the sidewall shape of the tire changed greatly, and the temperature rise in Figure 7 was faster, which led to the faster damage of the insert rubber.

## 4. Conclusions

According to the study, a successful attempt was made to employ simulation methods to study the key factors to be considered in the development of run-flat tires. The effects of heat and stress concentration on the zero-pressure durability of tires were studied by designing different components with different stiffness, heat generation and shape to change the stiffness of tire sidewall. The results revealed that the tensile strength and modulus of the insert compound decreased with the increase of temperature. The heat generation of the insert rubber was not only dependent on the loss factor, but also related to the deformation. By using the insert rubber with higher hardness or improving the structural stiffness of the tire, the deformation of the support rubber could be reduced and the heat storage of the support rubber could be reduced, which can significantly improve the sinking rate of the tire under zero pressure. In addition, the maximum strain energy density at the top of the bead and the maximum strain energy density of the cross-section insert rubber were significantly related to the durability of the zero pressure run flat tire and the initial failure point of the insert rubber. In conclusion, the balanced design of formula and structure was the key to tire development. At the same time, the potential of numerical simulation technology in tire development is verified. The application of these technologies could shorten the tire development process and improved the success rate of tire development.

## Figures and Tables

**Table 1:** Simulation model scheme.

List	Compound	Structure
Model-1	A-1	B-2
Model-2	A-2	B-2
Model-3	A-3	B-2
Model-4	A-4	B-2
Model-5	A-1	B-1
Model-6	A-2	B-1

(The compounds of A-1/A-2/A-3/A-4 will presented later on).

**Table 2:** Other structural designing elements of tires corresponding to structural analysis.

Structural Features		Model					
**Structural Designing Features**		1	2	3	4	5	6
**Insert Rubber**	compounds	A-1	A-2	A-3	A-4	A-1	A-2
**Belt**	materials	2 + 2*0.30HT-60EPD					
	Angle	29°					
**Carcass**	materials	1300D/2-110EPD					
	Carcass turn-up	low					
**Apex**	Shore A	79					
	height/mm	40					
**Bead**	materials	Φ1.295					
	arrange	4-5-6-5-4(φ466.8)					
**Charfer**	hardness	73					

Note: EPD, Ends per DM.

**Table 3:** The compound compounds (unit: parts per hundred rubber; phr).

Ingredient	A-1	A-2	A-3	A-4
TSR20	30	80	30	30
BR9000	70	20	70	70
N330	70	-	-	-
N550	-	65	60	50
ZnO	8	4	4	4
Stearic acid	2	1	1	1
TDAE	2	-	-	-
6PPD	1.5	3	3	3
TMQ	1	1	1	1
TBBS	2	2.3	2	2
TIBTD	0.8	-	-	-
HSOT-20	2.8	3	3	3

**Table 4:** Mixing procedure.

Stage	Mixer Operation	Time/s	Ram Press/bar	Mix Temp/°C	Rotor Speed/rpm
MB1	+Rubber and add.	15	4	-	55
	+CB	20	4	115	50
	+Oil	15	4	145	45
	Mix	30	4	165	40
	Drop	10	4	-	45
MB2	+MB1 and CB	20	4	125	40
	Mix	60	4	155	35
	Drop	15	4	-	40
FB	+MB2 and add	30	4	-	25
	Mix	30	4	-	25
	Mix	60	4	102	25
	Drop	15	4	-	25

MB = master batch; FM = final mixing; CB = carbon black; add = additives

**Table 5:** Hardness, tensile strength, and elongation at break at varying temperatures.

	A-1	A-2	A-3	A-4	A-1	A-2	A-3	A-4
**Hardness**	78	75	72	68	-	-	-	-
**Temperature/°C**	Tensile strength/MPa				Elongation at break/%			
**23**	13.08	18.85	13.59	10.69	131	230	222	225
**30**	13.67	18.74	14.12	12.49	151	231	240	237
**40**	12.55	18.7	12.66	10.95	134	247	216	230
**60**	10.25	11.17	10.51	10.38	121	164	177	220
**80**	9.65	10.88	8.99	8.30	118	174	152	175
**100**	8.46	11.71	7.86	7.85	102	179	139	175
**120**	8.25	7.32	6.77	6.72	102	115	122	140
**140**	7.05	8.12	6.49	6.24	91	134	112	136

**Table 6:** Dynamic modulus (E’) and loss factor (tan δ) of the vulcanizates at various temperatures.

Screening Temperature (°C)	A-1	A-2	A-3	A-4	A-1	A-2	A-3	A-4
	Loss Factor (tan δ)				E’(MPa)			
**40**	0.102	0.090	0.033	0.026	27.0	16.2	11.1	8.7
**60**	0.103	0.078	0.029	0.025	23.4	14.8	11.0	8.7
**80**	0.093	0.064	0.027	0.016	20.3	13.8	11.0	8.8
**100**	0.080	0.053	0.019	0.015	18.5	13.2	11.1	8.8
**120**	0.067	0.051	0.020	0.016	17.5	12.9	11.2	9.0
**140**	0.058	0.043	0.020	0.014	17.1	12.9	11.5	9.3

**Table 7:** Summary of the analytical and quantitative results of the groups of analysis models.

Model	Model 1	Model 2	Model 3	Model 4	Model 5	Model 6
**Maximum stress distribution of tire section/MPa**	4.405	5.777	3.893	3.617	4.065	4.063
**Maximum stress of bead apex and insert rubber of tire section/MPa**	1.924	3.179	2.822	3.322	1.662	1.744
**Maximum strain energy density of bead apex and insert rubber of tire section J/m^3^**	3.420	4.944	4.965	5.577	3.000	3.060

**Table 8:** Conventional static load test.

List	T-1	T-2	T-3	T-4	T-5	T-6
**Static radius under load/mm**	319	316	315	316	318	317
**Sinkage/mm**	18.9	20.9	20.6	20.9	18.6	20.0
**Sinkage/%**	17.4	19.3	19.2	19.4	17.2	18.5
**Imprint long axis/mm**	200	200	201	203	199	200
**Imprint minor axis/mm**	133	135	151	145	141	144

**Table 9:** Zero pressure static load test.

List	T-1	T-2	T-3	T-4	T-5	T-6
**Static radius under load/mm**	311	309	307	307	314	310
**Sinkage/mm**	23.2	25.0	27	27	21.5	24.4
**Sinkage/%**	21.9	23.7	25.6	25.6	20.0	23.0
**Imprint long axis/mm**	222	231	250	247	219	232
**Imprint minor axis/mm**	215	217	218	219	215	216

**Table 10:** Durability test results at zero pressure.

Tyre	Compound	Structure	Durability at Zero Pressure
T-1	A-1	B-2	57 min
T-2	A-2	B-2	54 min
T-3	A-3	B-2	44 min
T-4	A-4	B-2	39 min
T-5	A-1	B-1	3 h
T-6	A-2	B-1	1 h 50 min

## Data Availability

The data was basically presented in the article.
